# Climatography of the Salt River Valley Region of Arizona

**Published:** 1898-11

**Authors:** 


### Climatography of the Salt River Valley Region of Arizona, the Land of Health and Sunshine. By William Lawrence Woodruff, M. D., of Phœnix, Arizona. Chicago. R. R. Donnelley & Sons Co., Plymouth Place and Polk Street. Price, paper, 25 cents. Cloth, 50 cents.

This little book is a reprint of an article by Dr. Woodruff which first appeared in *The Hahnemannian Monthly* for December, 1895, was reprinted in *The Scientific American Supplement* for January, 1896, also in *The Sanitarian* for May, 1896, and *The Arizonian* for January, 1896. The book is intended, as stated on the title-page, for physicians and laymen and as a fund of information for the invalid and the home seeker.

The particular section of country of which it treats is claimed to be the most healthful in the world. The climate appears to be reasonably dry, the record for humidity averaging only thirty per cent. and sometimes far below that point. In this salubrious climate fevers and bowel troubles among infants during hot weather are almost unknown. Sunstroke is unknown. People suffering from insomnia and nervous prostration are greatly benefited.

The valley has everything that goes to make up a perfect winter home. It has the minimum of rainfall, 7 inches per annum; the minimum of atmospheric moisture, 30 per cent.; the minimum of air movement, 2½ miles per hour, and that generally from the southwest; the minimum death rate, 8.11 per 1,000 inhabitants, the minimum of malaria, there being none; low altitude, 1,100 feet above the sea level and maximum of sunshine.

It has all the merits of Egypt and Italy with none of their drawbacks. It has all that Florida enjoys without her moist, sticky atmosphere and malaria. It has the same balmy air and even temperature as California without her fogs, dampness, and malaria. It has the dry, bracing air of Colorado without her blizzards and high altitudes.

"In this favored spot, the sun-kissed valley of Salt River, you will find a haven of rest and safety for the invalid that fills all requirements, and the like of which does not exist in any other portion of the known world."

Tables of temperature are given and charts of isothermal lines. Thus the physician who desires to send his patient to a favorable climate has in this little volume a handy and reliable guide, the advice of which he can take without fear of making a mistake.

